# Prevalence and risk factors of *Acinetobacter baumannii* infection in Pediatric Intensive Care Unit at Thammasat University Hospital

**DOI:** 10.12688/f1000research.157612.1

**Published:** 2024-10-24

**Authors:** Pornumpa Bunjoungmanee, Kornkamon Rattanapan, Yamonbhorn Neamkul, Auchara Tangsathapornpong, Narissara Mungkornkaew, Prapasri Kulalert

**Affiliations:** 1 Department of Pediatrics, Faculty of Medicine, Thammasat University, Amphoe Khlong Laung, Pathum Thani, 12120, Thailand; 2 Head of Microbiology Laboratory, Thammasat University Hospital, Amphoe Khlong Luang, Pathum Thani, 12120, Thailand; 3 Department of Clinical Epidemiology, Faculty of Medicine, Thammasat University, Amphoe Khlong Luang,, Pathum Thani, 12120, Thailand

**Keywords:** Acinetobacter baumannii infection (ABI), pediatric intensive care unit (PICU), carbapenem-resistant Acinetobacter baumannii (CRAB)

## Abstract

**Background:**

*Acinetobacter baumannii* infection (ABI) is a concerning worldwide public health matter with high levels of morbidity and mortality, particularly in critically ill patients. This study aims to assess the prevalence, risk factors, and clinical outcomes of ABI in the pediatric intensive care unit (PICU) setting.

**Methods:**

A retrospective review was performed on pediatric patients admitted to the PICU over an 8-year period. Demographic characteristics, infection risk factors, and clinical outcomes were compared and analyzed between patients with ABI, determined to be the case group, and patients without ABI, determined to be the control group. The study also assessed the prevalence of ABI and its antimicrobial resistance profile.

**Results:**

Between June 2014 and May 2022, a total of 82 cases of ABI were identified, resulting in an overall prevalence of 5.02%. After applying the exclusion criteria, 12 cases were excluded. Consequently, 70 ABI cases in total and 140 cases in a control group were included in the study. Multivariable conditional logistic regression analysis identified chronic respiratory disease, mechanical ventilation lasting 3 days or more, and the use of piperacillin/tazobactam within the last 2 weeks as independent risk factors associated with ABI. The rate of carbapenem-resistant *A. baumannii* (CRAB) was notably high at 93.22%. Cases of ABI were associated with higher mortality rates and prolonged hospitalization compared to non-ABI cases.

**Conclusion:**

ABI remains a critical pathogen in the PICU. The presence of chronic respiratory disease, use of mechanical ventilation for at least three days, and a history of receiving piperacillin/tazobactam within the last 2 weeks are significant risk factors for ABI. The high level of antibiotic resistance, especially to carbapenems, highlights the emphasis for more stringent infection control practices and the creation of novel antimicrobial therapies.

## Introduction

*Acinetobacter baumannii* causes significant problematic issues in healthcare-associated infections (HAIs). *A. baumannii* usually causes various infections, including sepsis, pneumonia, meningitis, urinary tract infection, skin and soft tissue infection. ^1– 3^ The critical factors contributing to the global challenge of *A. baumannii* in healthcare settings include the pathogen’s capacity to rapidly evolve mechanisms of drug resistance, its ability to persist in the environment, leading to widespread transmission, and the high morbidity and mortality rates of infections. ^4– 7^ The emergence of pan-antibiotic-resistant *A. baumannii* and the lack of effective treatments have led to its classification as a serious public health concern. *A. baumannii* has been designated as an urgent threat, necessitating the development of enhanced therapeutic strategies. ^8, 9^

Previous studies demonstrated the emergence of *A. baumannii* infections (ABI) with extremely high rates of carbapenem resistance among adults in Thailand, ranging between 70% to 80%, and mortality rates exceeding 60%. ^10– 12^ Consequently *,* ABI is estimated to result in over 15,000 deaths each year. ^13^ However, there is limited research on *A. baumannii* in pediatric populations, particularly within intensive care unit (ICU) settings. This study aimed to identify the prevalence and potential risk factors of ABI, along with the outcomes in critically ill children.

## Methods

### Ethic statement

This research study received approval from the Human Research Ethics Committee of Thammasat University (Medicine), in accordance with international guidelines, including the Declaration of Helsinki, The Belmont Report, CIOMS Guidelines, and the International Conference on Harmonization-Good Clinical Practice (ICH-GCP). The approval number was MTU-EC-PE-0-125/65 on September 2, 2022. Data collection commenced following the Ethics Committee’s authorization. Informed consent was waived due to the retrospective nature of the study. Data was then collected through the review of patient medical records without any direct participant contact or intervention.

### Study design

The study was a retrospective case-control study of pediatric patients under the age of 15 years at Thammasat University Hospital, a tertiary care facility with an 8-bed pediatric intensive care unit (PICU), from June 2014 and May 2022. All strains of *A. baumannii* were classified as causative agents of HAIs based on identification in the microbiology laboratory database, meeting the established criteria for significant pathogens.

To identify cases of *A. baumannii* from PICU, we reviewed culture results from specimens obtained throughout the patients’ hospitalizations. Cases were defined as patients who acquired ABI 48 hours or more following admission to the PICU. Controls were defined as patients admitted to the PICU without ABI. Seventy cases and 140 controls were randomly matched in a 1: 2 ratio. Controls were randomly selected from the same month as cases admitted to the PICU. Patients with *A. baumannii* identified within the first 48 hours of PICU admission, a PICU stay of less than 48 hours, or incomplete records were excluded.

*A. baumannii* was identified and confirmed from the sample using standard microbiological techniques. The susceptibility of the isolates was assessed by determining the minimum inhibitory concentrations following the Clinical and Laboratory Standards Institute (CLSI) guidelines, ^14^ using the VITEK2 compact system (bioMérieux, France). Colistin susceptibility was assessed using broth microdilution in accordance with CLSI ^14^ and The European Committee on Antimicrobial Susceptibility Testing (EUCAST) guidelines. ^15^ Multidrug-resistant *A. baumannii* (MDRAB) is defined as isolates exhibiting resistance to at least one agent from three or more antimicrobial classes. Extensively drug-resistant *A. baumannii* (XDRAB) refers to isolates that demonstrate susceptibility exclusively to tigecycline and colistin, while exhibiting resistance to all other antibiotic classes. Carbapenem-resistant *A. baumannii* (CRAB) is characterized by nonsusceptibility involving at least one of three agents within the carbapenem class. ^16^

*Data collection*

This study utilized a systemic computer-assisted database search to obtain retrospective data on patients discharged from the PICU. Information on demographics, clinical outcomes, and microbiological data were gathered from medical and laboratory records.

*Data analysis*

Categorical variables were presented as frequencies and percentages, whereas continuous variables were given as mean and standard deviation. Non-normally distributed data was expressed as median and interquartile range (IQR). The Rank-Sum test was utilized for non-normally distributed continuous variables and the t-test was applied for comparing normally distributed continuous variable. Chi-square or Fisher’s exact test was utilized to compare categorical data as appropriate. The identification of independent factors associated with ABI was analyzed using univariable and multivariable conditional logistic regression, with statistical significance set at p < 0.05. Statistical analysis was done using STATA version 17.0.

## Results

Throughout the 8-year duration of the study, a total of 1,635 PICU admission were recorded. Eighty-two cases of ABI were identified giving the overall prevalence of 5.02%. Twelve cases were excluded (7 cases with *A. baumannii* identified within the first 48 hours, and 5 cases had incomplete medical data). The remaining ABI were 70 cases. Among them, the percentage of CRAB reached 93.22%. One hundred forty patients with negative *A. baumannii* specimens who met the matching criteria were included as controls.

Upon analyzing the annual prevalence of ABI in the PICU, the rate exhibited a fluctuating pattern from 2014 to 2022. From 2014 to 2016, the rate remained stable at 3.00, followed by a noticeable increase in 2017 to 4.14. The prevalence peaked in 2018 at 5.94, before slightly declining to 5.22 in 2019. A more significant decrease was observed in 2020 and 2021, with rates dropping to 3.97 and 3.78, respectively. However, in 2022, the prevalence rebounded to 5.4, approaching the 2018 peak. This overall trend suggests periodic fluctuations in ABI, with notable peaks occurring in 2018 and 2022.

Patients’ clinical characteristics in each group are shown in Table 1. The ABI cases exhibited a younger age and a greater frequency of comorbidities, referral from another hospital, prior hospitalizations, antibiotic use, mechanical ventilation, and central/arterial line placements. Additionally, the use of broad-spectrum antibiotics and lengthy hospitalization prior to the onset of ABI tended to be more frequent in this group. Patients with ABI experienced prolonged hospitalization after the onset of infection and demonstrated an increasing mortality rate compared to patients without ABI.

**Table 1.** Baseline characteristics of children in pediatric intensive care unit, 2014-2022.

Characteristics	ABI case (n=70)	Non-ABI case (n=140)	*p*-value
Male, n (%)	41 (58.57)	77 (55.00)	0.623
Age, months, median (IQR)	8.5 (3, 46)	36.5 (11.5, 79.5)	<0.001 *
Age ≤12 months, n (%)	38 (54.29)	37 (26.43)	<0.001 *
Comorbidities, n (%)			
Neuromuscular disease	4 (5.71)	10 (7.14)	0.778
Chronic respiratory disease ^a^	22 (31.43)	9 (6.43)	<0.001 *
Congenital heart disease	34 (48.57)	46 (32.86)	0.027 *
Genetic disease	23 (32.86)	23 (16.43)	0.007 *
Immunodeficiency ^b^	11 (15.71)	16 (11.43)	0.382
Referral from another hospital (%)	16 (22.86)	9 (6.43)	0.001 *
Prior hospitalization within 3 months (%)	34 (48.57)	43 (30.71)	0.011 *
Prior antibiotic use within 3 months (%)	24 (34.29)	23 (16.43)	0.003 *
Broad-spectrum antibiotic ^c^ use within the last 2 weeks prior to development ABI, n (%)	70 (100)	110 (78.57)	<0.001 *
3 ^rd^ generation cephalosporin, n (%)	46 (65.71)	87 (62.14)	0.613
Piperacillin/tazobactam, n (%)	25 (35.71)	17 (12.14)	<0.001 *
Aminoglycoside, n (%)	10 (14.29)	14 (10.00)	0.357
Carbapenems, n (%)	31 (44.29)	40 (28.57)	0.023 *
Procedures within 2 weeks prior to development ABI			
Mechanical ventilation n (%)	63 (90.00)	75 (53.57)	<0.001 *
Duration of mechanical ventilation ≥ 3 days, n (%)	58 (82.86)	43 (30.71)	<0.001 *
Central line or arterial line placement, n (%)	62 (88.57)	96 (68.57)	0.002 *
Chest drain/Abdominal drain/Pericardial drain, n (%)	20 (28.57)	31 (22.14)	0.306
HD/ECMO, n (%)	6 (8.57)	5 (3.57)	0.186
Duration of PICU days prior to development of ABI, day (median, IQR)	8 (4, 16)	4 (3, 8)	<0.001 *
Duration of PICU days > 7 days prior to development of ABI, n (%)	41 (58.57)	47 (33.57)	0.001 *
Outcome			
In hospital mortality, n (%)	11 (15.71)	6 (4.29)	0.004 *
Length of hospital stays after *A. baumannii* isolated, days (IQR)	39.5 (25, 88)	13 (8, 20)	<0.001 *

Abbreviations: PICU, pediatric intensive care unit; ABI, *Acinetobacter baumannii* infection; non-ABI, non- *Acinetobacter baumannii* infection; IQR, interquartile range; HD, hemodialysis; ECMO, extracorporeal membrane oxygenation

^*^ Statistically significant.

^a^ Bronchopulmonary dysplasia (BPD), and asthma.

^b^ Hematologic malignancy, on immunosuppressive drug, primary immunodeficiency.

^c^ Third-generation cephalosporins, beta-lactam/beta-lactamase inhibitors, aminoglycosides, and carbapenems.

Among patients with ABI, there were 28 cases (40.00%) identified as ventilator-associated pneumonia, 10 cases (14.28%) as bloodstream infections, 8 cases (11.43%) as catheter-related bloodstream infections, 5 cases (7.15%) as skin and soft tissue infections, 2 cases (2.86%) as catheter-associated urinary tract infections, 2 cases (2.86%) as ventricular shunt infections, and 15 cases (21.42%) were identified as having a combination of multiple infection types.

In our study, ABI was associated with a mortality rate of 15.71%. All fatal cases involved patients with at least one comorbidity or a history of major surgical intervention. Of these, two patients had acute leukemia, two had chronic respiratory disease, two had an underlying genetic disorder with airway anomalies, two had congenital heart disease, two had a recent car accident and had undergone major surgery in the past 10 days, and one had a brain tumor with craniotomy in the past two weeks. All cases were complicated by septic shock and multiorgan failure, resulting in fatal outcomes.

Cultures were obtained from 70 cases of ABI. Of these 177 isolates, 133 were from respiratory samples (75.14%), 35 were from blood (19.77%), 5 were from pus (2.83%), and 4 were from urine (2.26%). High co-resistance rates were observed for meropenem (93.22%), imipenem (92.00%), piperacillin-tazobactam (90.81%), ciprofloxacin (89.60%), ceftazidime (88.57%), amikacin (84.09%), and cefoperazone-sulbactam (81.95%). In contrast, low resistance rates were observed for tigecycline (3.21%). All the isolates were susceptible to colistin according to the EUCAST guidelines, ^15^ while exhibiting intermediate susceptibility to colistin based on the CLSI guidelines. ^14^ According to the criteria established by Magiorakos et al, ^16^ most of the isolates were classified as MDRAB (n = 170, 96.05%) and 87.57% were classified as XDRAB (n = 155).

Factors associated with ABI, as determined by both univariable and multivariable conditional logistic regression analysis, are presented in Table 2. In the univariable analysis, the risk factors associated with ABI included being under 12 months of age, chronic respiratory disease, congenital heart disease, and genetic disease. Furthermore, referral from another hospital, prior hospitalization within 3 months, prior antibiotic use within 3 months, and PICU stay longer than 7 days were significantly associated with ABI. Mechanical ventilation lasting 3 days or more, central line or arterial line placement, using piperacillin/tazobactam or carbapenems within the last 2 weeks significantly increased the risk of ABI.

**Table 2.** Univariable and multivariable conditional logistic regression analysis of risk factors associated with *Acinetobacter baumannii* infection.

Characteristics	Univariable analysis			Multivariable analysis		
	Odds ratio	95% CI	p-value	Odds ratio	95% CI	p-value
Age under 12 months	3.65	1.89, 7.08	<0.001 *			
Chronic respiratory disease ^a^	5.32	2.37, 12.00	<0.001 *	5.13	1.31, 20.11	0.019 *
Congenital heart disease	1.91	1.06, 3.45	0.031 *			
Genetic disorders	2.65	1.30, 5.38	0.007 *			
Referral from another hospital	5.30	1.91, 14.70	0.001 *			
Prior hospitalization within 3 months	2.38	1.24, 4.57	0.009 *			
Prior antibiotic use within 3 months	3.54	1.54, 8.12	0.003 *			
Piperacillin/tazobactam use within 2 weeks prior to development ABI	4.72	2.09, 10.66	<0.001 *	3.22	1.01, 10.36	0.049 *
Carbapenem use within 2 weeks prior to development ABI	1.95	1.07, 3.53	0.028 *			
Mechanical ventilation at least 3 days	11.35	4.81, 26.77	<0.001 *	9.47	2.86, 31.30	<0.001 *
Central line or arterial line placement	3.67	1.57, 8.58	0.003 *			
PICU stay longer than 7 days	2.95	1.56, 5.61	0.001 *			

Abbreviations: ABI, *Acinetobacter baumannii* infection; 95% CI, 95% confident interval; PICU, pediatric intensive care unit.

^*^ Statistically significant.

^a^ Bronchopulmonary dysplasia (BPD), and asthma.

The final multivariable conditional logistic regression analysis identified chronic respiratory disease, mechanical ventilation lasting 3 days or more, and the use of piperacillin/tazobactam within the last 2 weeks as independent factors associated with ABI.

## Discussion

*A. baumannii* species are emerging as a significant public health threat, frequently exhibiting multidrug resistance and being associated with high mortality rates. An analysis of data collected over eight consecutive years revealed that the overall prevalence of ABI in the PICU was approximately 5.02%. This prevalence is lower than previous studies, which ranged from 8.30% to 15.30%. ^17– 19^

The prevalence of ABI significantly declined between 2020 and 2021, a period coinciding with the global COVID-19 pandemic. Several studies have reported reductions in ABI rates during this time, with decreases ranging from 1.00% to 3.00%. ^20– 23^ This reduction is likely associated with widespread lifestyle changes and the emphasis for more stringent practices of infection control aimed at timing the transmission of SARS-CoV-2. These measures contributed to a decline in respiratory infections among children, reduced ICU admissions for non-COVID-19 cases, a reduction in hospitalization rates, and fewer pediatric patients with severe SARS-CoV-2 infections who required PICU admission.

Similar to adults, the major factors related to the emergence and spread of ABI in children, as demonstrated in previous studies, included misguided antimicrobial treatment and prior invasive procedure such as mechanical ventilation, central venous catheterization, and urinary catheters. ^1, 24– 26^ Our study found that the use of piperacillin-tazobactam, prolonged mechanical ventilation for a minimum of 72 hours, and chronic respiratory disease were significantly associated with an increased risk of ABI.

Nowadays, where carbapenems are frequently administered, particularly for the treatment of severe cases, the selective pressure may encourage the emergence and spread of CRAB. As a result, the widespread administration of carbapenems is a potential risk factor of ABI. ^6, 27– 29^ Differing from previous studies, our study identified piperacillin-tazobactam as a significant risk factor. This difference was attributed to the antibiotic prescribing practices at Thammasat University Hospital.

Piperacillin-tazobactam was widely available and could be prescribed by physicians across various specialties without restriction. Conversely, the use of carbapenem was tightly regulated, requiring authorization from a pediatric infectious disease specialist prior to prescription. As a result, physicians commonly chose piperacillin-tazobactam, a broad-spectrum antibiotic with a similar efficacy profile to carbapenems, particularly for critically ill patients in the PICU. This prescribing pattern likely contributed to the observed association between piperacillin-tazobactam and an increased risk of ABI, which was statistically significant in this study.

Our study is the first to identify chronic respiratory disease, including bronchopulmonary dysplasia (BPD) and asthma, as significant risk factors for ABI. In these patients, the compromised pulmonary architecture and function facilitated bacterial colonization and subsequent infection. Furthermore, these patients often had a history of recurrent hospitalizations and a potential need for mechanical ventilation, both of which contribute to an increased risk of hospital-acquired ABI. Research data in adults demonstrated that chronic obstructive pulmonary disease (COPD) was a significant risk factor for acquiring ABI. ^30– 33^ These findings underscore the significance of chronic lung conditions in predisposing individuals to ABI across different age groups.

Our study demonstrated that mechanical ventilation lasting 3 days or more was an independent factor associated with ABI. Encouraging non-invasive mechanical ventilation and gradually weaning respiratory support while assessing the necessity for endotracheal intubation, ^34^ combined with the implementation of targeted antibiotic stewardship program and the reinforcement of rigorous infection control protocols within healthcare setting, ^5^ can reduce the hospital-acquired pneumonia/ventilator-associated pneumonia (HAP/VAP) and substantially mitigate the risk of ABI.

The rise of CRAB poses a significant challenge to treatment, as therapeutic options become markedly constrained. These resistant strains are associated with higher morbidity and mortality rates and are increasingly recognized as among the most problematic bacterial pathogens. ^1, 3, 6, 7, 29, 35^ In our study, the prevalence of CRAB reached 93.22%. The presence of CRAB strains is frequently associated with outbreaks in hospital ICU and presents significant challenges to infection control within healthcare settings. ^5, 36, 37^ To minimize the risk, carbapenems should be used judiciously, and infection control measures should be strictly implemented.

The high mortality rate associated with ABI may be related to the extent of antimicrobial resistance, the efficacy of empirical therapy, and the availability of targeted therapeutic options. The overall mortality rate in our study was 15.71%, consistent with finding from previous studies. ^3, 28, 38^ However, other studies reported higher mortality rate, ranging from 28.3% to 48.65%. This variation is likely attributable to the inclusion of patients with *A. baumannii* sepsis, with or without septic shock, and delayed in initiating appropriate antibiotic regimens effective against *A. baumannii* once the infection was established. ^1, 4, 6, 29, 39^

*A. baumannii* is an important threat to patients of all ages, including children. Therefore, ongoing surveillance of ABI in pediatric populations and continuous assessment of effective prevention strategies for at-risk children are essential. The widespread prevalence of antibiotic-resistant *A. baumannii* strains, combined with the scarcity of new antimicrobial agents, restricts the available therapeutic options. Prioritizing vaccine development against *A. baumannii* may be regarded as a potential strategy to combat the challenge of antimicrobial resistance in this pathogen moving forward, which is currently under investigation in research studies. ^40– 43^

### Strengths and limitations

The study demonstrates several notable strengths. The extended study period of eight years enables a comprehensive analysis of trends in ABI over time. Focusing on the critically ill pediatric population addresses a significant knowledge gap in understanding the risk factors associated with ABI through multivariable conditional logistic regression analysis and highlights the patterns of antibiotic resistance.

The study had some limitations that should be considered. The retrospective design introduces potential biases. Additionally, being a single-center study limits the generalizability of the findings to other healthcare settings with differing patient demographics and hospital practices. Furthermore, the study lacks detailed genetic analysis, thereby limiting the depth of understanding regarding the genetic mechanisms behind antibiotic resistance.

## Conclusion

This study highlights the significant burden of ABI, particularly carbapenem-resistant strains, in the PICU setting. Prolonged mechanical ventilation for at least 3 days, underlying chronic respiratory disease, and the administration of piperacillin/tazobactam contribute to a higher risk of developing ABI. The findings of our study underscore the importance of the stringent practices of infection control and antibiotic stewardship measures, alongside advocating the use of non-invasive mechanical ventilation in lieu of endotracheal intubation, which can substantially mitigate the risk of ABI. Although the study identifies key risk factors and provides valuable insights into the epidemiology of *A. baumannii* in the PICU, further research and enhanced infection prevention strategies are necessary to reduce morbidity and mortality related to ABI in critically ill pediatric populations.

## Data Availability

Zenodo: Prevalence and risk factors of *Acinetobacter baumannii* infection in Pediatric Intensive Care Unit at Thammasat University Hospital. The anonymized data sets of this project are available in the Zenado: https://doi.org/10.5281/zenodo.13921141. ^44^ The project contains the following underlying data:
•
Data patient case reviwed.xlsx
•
Data patient control reviewed.xlsx Data are available under the term of the Creative Commons Attribution 4.0 International license (CC-BY 4.0). Zenodo: Prevalence and risk factors of *Acinetobacter baumannii* infection in Pediatric Intensive Care Unit at Thammasat University Hospital: DOI: https://doi.org/10.5281/zenodo.13921141. ^44^ The project contains the following checklist:
• STROBE checklist for prevalence and risk factors of *Acinetobacter baumannii* infection in pediatric intensive care unit at Thammasat university hospital Data are available under the term of the Creative Commons Attribution 4.0 International license (CC-BY 4.0)
