# Supplementary material for: Prevalence and risk factors of Acinetobacter baumannii infection in Pediatric Intensive Care Unit at Thammasat University Hospital
Source: F1000Res. 2025 May 20;13:1269. Originally published 2024 Oct 24. [Version 3] doi: 10.12688/f1000research.157612.3 (PMC12125569; doi:10.12688/f1000research.157612.3)
Supplement: Peer review history

# Reviewer report: Reviewer response for version 3

**DOI:** 10.5256/f1000research.182195.r386111

**Published:** 2025-05-30

**Authors:** Noppadol Wacharachaisurapol

**Affiliations:** 1 Chulalongkorn University, Bangkok, Thailand

Thank you for the revised manuscript and detailed responses. I am satisfied with the authors' clarifications and revisions. I have no further comments or suggestions.

Is the work clearly and accurately presented and does it cite the current literature?

Partly

If applicable, is the statistical analysis and its interpretation appropriate?

I cannot comment. A qualified statistician is required.

Are all the source data underlying the results available to ensure full reproducibility?

Yes

Is the study design appropriate and is the work technically sound?

Yes

Are the conclusions drawn adequately supported by the results?

Yes

Are sufficient details of methods and analysis provided to allow replication by others?

Partly

Reviewer Expertise:

Pediatric infectious diseases; antimicrobial pharmacokinetics

I confirm that I have read this submission and believe that I have an appropriate level of expertise to confirm that it is of an acceptable scientific standard.

# Reviewer report: Reviewer response for version 2

**DOI:** 10.5256/f1000research.181112.r381841

**Published:** 2025-05-13

**Authors:** Noppadol Wacharachaisurapol

**Affiliations:** 1 Chulalongkorn University, Bangkok, Thailand

Article: “Prevalence and risk factors of Acinetobacter baumannii infection in Pediatric Intensive Care Unit at Thammasat University Hospital”

 Thank you for the revised manuscript and your detailed responses. I have a few additional comments and suggestions for your consideration:

**Methods**

- Please clarify how tigecycline susceptibility was evaluated, given that there are no established MIC breakpoints for *A. baumannii* from either CLSI or EUCAST.

**Results**

 Table 1

- It remains unclear how data were obtained for the non-ABI group, considering that no ABI events occurred in this group. Kindly clarify in the Methods section how the following characteristics were defined or collected:
  - Broad-spectrum antibiotic use within the last 2 weeks **prior to the development of ABI**
  - Procedures within 2 weeks **prior to the development of ABI**
  - Duration of PICU days **prior to the development of ABI**
  - Duration of PICU days > 7 days **prior to development of ABI**, n (%)
    - Additionally, please reconsider the use of the term “duration” here, as the result is presented as n (%). If you are referring to the proportion of patients who had PICU stays longer than 7 days before developing ABI, please revise the wording accordingly.
  - Length of hospital stays **after *A. baumannii* isolated**
    - The results were 40.5 days (ABI group) and 24 days (non-ABI group). Please clarify whether these results represent the total length of hospital stay, which I believe they do, or only the duration following *A. baumannii* isolation.

Is the work clearly and accurately presented and does it cite the current literature?

Partly

If applicable, is the statistical analysis and its interpretation appropriate?

I cannot comment. A qualified statistician is required.

Are all the source data underlying the results available to ensure full reproducibility?

Yes

Is the study design appropriate and is the work technically sound?

Yes

Are the conclusions drawn adequately supported by the results?

Yes

Are sufficient details of methods and analysis provided to allow replication by others?

Partly

Reviewer Expertise:

Pediatric infectious diseases; antimicrobial pharmacokinetics

I confirm that I have read this submission and believe that I have an appropriate level of expertise to confirm that it is of an acceptable scientific standard, however I have significant reservations, as outlined above.

# Reviewer report: Reviewer response for version 2

**DOI:** 10.5256/f1000research.181112.r381842

**Published:** 2025-05-05

**Authors:** Visal Moolasart

**Affiliations:** 1 Department of Disease Control, Bamrasnaradura Infectious Diseases Institute, Ministry of Public Health, Nonthaburi, Thailand

I am satisfied with the revised version of the manuscript and acknowledge the limitations of this study, including its retrospective design, single-center setting, and small sample size.

Is the work clearly and accurately presented and does it cite the current literature?

Partly

If applicable, is the statistical analysis and its interpretation appropriate?

Partly

Are all the source data underlying the results available to ensure full reproducibility?

Yes

Is the study design appropriate and is the work technically sound?

Partly

Are the conclusions drawn adequately supported by the results?

Partly

Are sufficient details of methods and analysis provided to allow replication by others?

Partly

Reviewer Expertise:

infectious, HIV EID IPC and children

I confirm that I have read this submission and believe that I have an appropriate level of expertise to confirm that it is of an acceptable scientific standard.

# Reviewer report: Reviewer response for version 1

**DOI:** 10.5256/f1000research.173078.r365254

**Published:** 2025-02-18

**Authors:** Noppadol Wacharachaisurapol

**Affiliations:** 1 Chulalongkorn University, Bangkok, Thailand

Article: "Prevalence and risk factors of Acinetobacter baumannii infection in Pediatric Intensive Care Unit at Thammasat University Hospital"

 This work provides valuable information on the prevalence, risk factors, and clinical outcomes of ABI in the PICU setting. The paper is well organized; however, some clarifications may improve it.

 SPECIFIC RECOMMENDATIONS

**Introduction**

 Page 3:

- The authors mention limited research on *A. baumannii* infections in pediatric ICUs, but some studies exist. For context, it would be helpful to summarize previously reported risk factors and clinical outcomes in adults and/or in PICU settings.

**Methods**

 Page 3:

- Controls were defined as patients admitted to the PICU without ABI.
  - Please describe any matching criteria used for controls.
  - Please clarify the indications for PICU admission in controls to ensure comparability with cases. For instance, whether controls had other MDRO infections or were admitted for non-infectious conditions, as patients admitted solely for postoperative monitoring may have a lower mortality risk.

**Results**

 Page 4-5: Table 1

- Several characteristics (e.g., broad-spectrum antibiotic use within the last 2 weeks) are described as occurring "prior to ABI development." For controls, prior to what event were these characteristics assessed regarding no ABI in this group?
- Please clarify in the methods the rationale for selecting a 2-week cutoff for characteristics like antibiotic use and procedures.
- Please include the total number (%) of patients with comorbidities to provide an overall perspective before listing specific conditions.
- The denominators for mechanical ventilation ≥ 3 days appear to be 70 and 140, though they likely represent subgroups of ventilated patients. If so, the denominators should be 63 and 75 (only those on mechanical ventilation) to ensure accurate proportions.

Page 5:

- The study states that tigecycline resistance was low (3.21%), but CLSI and EUCAST do not provide breakpoints for this drug. Please clarify how resistance was determined or which breakpoint was used.

Is the work clearly and accurately presented and does it cite the current literature?

Partly

If applicable, is the statistical analysis and its interpretation appropriate?

I cannot comment. A qualified statistician is required.

Are all the source data underlying the results available to ensure full reproducibility?

Yes

Is the study design appropriate and is the work technically sound?

Yes

Are the conclusions drawn adequately supported by the results?

Yes

Are sufficient details of methods and analysis provided to allow replication by others?

Partly

Reviewer Expertise:

Pediatric infectious diseases; antimicrobial pharmacokinetics

I confirm that I have read this submission and believe that I have an appropriate level of expertise to confirm that it is of an acceptable scientific standard, however I have significant reservations, as outlined above.

# Response

**Published:** 2025-04-24

**Authors:** Pornumpa Bunjoungmanee

**Affiliations:** pediatric, Thammasat University, Pathum Thani, Thailand

 Thank you Dr. Noppadol Wacharachaisurapol for your valuable time and interest for reviewing

 this work and for the detailed feedback and recommendations for improving this manuscript.

 We have pasted your comments below, in bold, with responses in turn.

  

1. The authors mention limited research on *A. baumannii* infections in pediatric ICUs, but some studies exist. For context, it would be helpful to summarize previously reported risk factors and clinical outcomes in adults and/or in PICU settings.

**     Ans**

**A comprehensive overview of the risk factors for *Acinetobacter baumannii* infection in both adult and pediatric populations has been provided in the background section.**

       2. Page 3

           : Please describe any matching criteria used for controls.

**Ans**

**In the initial version of the article, cases and controls were selected from patients admitted to the pediatric intensive care unit (PICU) during the same period, with a minimum stay of 48 hours. Random matching was conducted in a 1:2 ratio. The exclusion criteria included pediatric patients whose culture reported grew *Acinetobacter baumannii* within 48 hours of PICU admission or those with incomplete medical records. However, this approach posed a risk of potential confounding.**

**To address this issue, additional exclusion criteria were included: a minimum Pediatric Logistic Organ Dysfunction-2 (PELOD-2) score of 10 and an extended minimum PICU stay of 120 hours. These modifications were implemented to reduce confounding in the selection of case and control groups. A PELOD-2 score of ≥10 is considered a medium score, and is associated with an estimated mortality risk of approximately 10%. ^1-2 ^The researchers consider this threshold appropriate for identifying patients eligible to participate in the study.**

**Reference **

1. **Leteurtre S, Duhamel A, Salleron J, et al. PELOD-2: An Update of the PEdiatric Logistic Organ Dysfunction Score. Crit Care Med. 2013;41(7):1761-73. **
2. **López MPA, Prata-Barbosa A, Lima-Setta F. Severity of illness scores in the pediatric intensive care unit: a practical guide. Crit Care Sci. 2024 Oct 11;36:e20240205en.**
:

Please clarify the indications for PICU admission in controls to ensure comparability with cases. For instance, whether controls had other MDRO infections or were admitted for non-infectious conditions, as patients admitted solely for postoperative monitoring may have a lower mortality risk.

****

**Ans**

**The exclusion criteria were modified to mitigate confounding factors resulting from the selection of an inappropriate control group. As a result, an additional criterion was implemented: all study participants must have a PELOD score of 10 or higher upon admission to the intensive care unit and a minimum PICU admission duration of 120 hours.**

      3. Table 1

           : Several characteristics (e.g., broad-spectrum antibiotic use within the last 2 weeks) are described as occurring "prior to ABI development." For controls, prior to what event were these characteristics assessed regarding no ABI in this group?

**Ans**

**In the control group, the researchers collected data on antibiotic administration throughout the patient's hospitalization in the intensive care unit during the 2 weeks of the patient's admission to the PICU. If the patient’s stay exceeded 2 weeks, antibiotic data was recorded based on the antibiotics received within 2 weeks prior to transfer out of the ICU.**

             : Please clarify in the methods the rationale for selecting a 2-week cutoff for characteristics like antibiotic use and procedures.

**Ans**

**Previous studies have mostly not specified the duration of antibiotic administration or procedures prior to the acquisition of *Acinetobacter baumannii* infection or its correlation. They generally only mention a history of receiving broad-spectrum antibiotics or undergoing procedures. Therefore, the researchers aim to investigate and compare the duration of broad-spectrum antibiotic use or procedures performed before the risk of *Acinetobacter baumannii* infection increases. A 2-week cutoff is based on previous studies indicating that the use of broad-spectrum antibiotics or procedures performed for approximately 1-2 weeks is associated with an increased risk of *Acinetobacter baumannii* infection**.

**Reference**

1. **Xu, N. N., Wang, H., and Zhou, M. (2019a). Analysis of risk factors and prevention strategies of extensive drug- resistant Acinetobacter baumannii infection and colonization in patients with mechanical ventilation.  J. Hosp. Infect. 92, 47–53.**
2. **Shihan Zhang, et al. An integrative review on the risk factors, prevention, and control strategies for carbapenem-resistant Acinetobacter baumannii colonization in critically ill patients. Front Microbiol 2023.10:15:1519906. **
3. **Beavers SF, et al. Comparison of risk factors for recovery of Acinetobacter baumannii during outbreaks at two Kentucky hospitals, 2006. Public Health Rep. 2009 Nov-Dec;124(6):868-74.**
4. **Hong Y, et al. Initial indicators for the prognosis of *Acinetobacter baumannii* bacteremia in children. BMC Infect Dis. 2023 Sep 29;23(1):640.  **

            : Please include the total number (%) of patients with comorbidities to provide an overall perspective before listing specific conditions.

**Ans**

**The information has been updated following the recommendations**

             : The denominators for mechanical ventilation ≥ 3 days appear to be 70 and 140, though they likely represent subgroups of ventilated patients. If so, the denominators should be 63 and 75 (only those on mechanical ventilation) to ensure accurate proportions.

**Ans**

**The information has been updated following the recommendations.**

           4. Page 5

              : The study states that tigecycline resistance was low (3.21%), but CLSI and EUCAST do not provide breakpoints for this drug. Please clarify how resistance was determined or which breakpoint was used.

**Ans**

**Due to the lack of established guidelines for interpreting tigecycline susceptibility against *Acinetobacter baumannii*, the laboratory currently utilizes the CLSI criteria for *Enterobacteriaceae* as an alternative for interpretation same as the previous studies.**

**Reference**

1. **Kim D, et al. The Changes in Epidemiology of Imipenem-Resistant Acinetobacter baumannii Bacteremia in a Pediatric Intensive Care Unit for 17 Years. J Korean Med Sci. 2022 Jun 8;37(24):e196.**
2. **Bejarano JIC, et al. Carbapenem-Resistant Acinetobacter baumannii Infection in Children From a Third-Level Hospital in Mexico: Clinical Characteristics and Molecular Epidemiology. J Pediatric Infect Dis Soc. 2023 Jul 31;12(7):431-435**
3. **Zhu Y, et al. Insight into carbapenem resistance and virulence of Acinetobacter baumannii from a children's medical centre in eastern China. Ann Clin Microbiol Antimicrob. 2022 Nov 5;21(1):47.**
4. **Fang C, er al. Epidemiology and Cytokine Levels among Children with Nosocomial Multidrug-Resistant Acinetobacter baumannii Complex in a Tertiary Hospital of Eastern China. PLoS One. 2016 Aug 31;11(8):e0161690. **

# Reviewer report: Reviewer response for version 1

**DOI:** 10.5256/f1000research.173078.r342361

**Published:** 2024-11-22

**Authors:** Visal Moolasart

**Affiliations:** 1 Department of Disease Control, Bamrasnaradura Infectious Diseases Institute, Ministry of Public Health, Nonthaburi, Thailand

This manuscript presents a compelling message and provides valuable insights into the "Prevalence and Risk Factors of *Acinetobacter baumannii* Infection in the Pediatric Intensive Care Unit at Thammasat University Hospital." However, there are several issues that merit further consideration.

 1. This case-control study compares the risk factors for *Acinetobacter baumannii* infections with those for non- *Acinetobacter baumannii* infections (control group). While the information provided is valuable, further consideration of several issues is necessary, such as the selection criteria and characteristics of the control group, potential confounding factors, and the robustness of the data analysis.

         -A study by Uwingabiye J et al reported that *Acinetobacter baumannii* accounted for 22.6% of infections in the ICU. In several study, the mortality rate associated with A. baumannii infections ranges from 26.5% to 91%, reaching as high as 70% for infections caused by resistant strains. These findings highlight the severity of cases involving A. baumannii.

**-** **The selection of a control group is a critical aspect of case-control study design**. In this study, the control group was defined as patients with non- *Acinetobacter baumannii* infections, representing less severe cases with fewer underlying conditions. To address this issue, the authors might consider selecting a more appropriate control group for comparison. For example, other studies have compared children with carbapenem-resistant A. baumannii (CRAB) infections to those with carbapenem-susceptible A. baumannii (CSAB) infections, which could provide a more balanced and clinically relevant comparison.

        The authors may consider matching the control group by adding specific conditions to ensure appropriate integration and analysis. This approach could help create a more balanced comparison and improve the validity of the findings.

  2. **Case group** : The *Acinetobacter baumannii* group is divided into two subgroups: resistant and non-resistant strains. Please consider this distinction, as several factors may differ between these subgroups and could influence the analysis and outcomes.

 3. If the authors intend to compare two groups for factor analysis, they should consider the sample size in each group to ensure sufficient statistical power and reliable results.

 4. This information demonstrated” Upon analyzing the annual prevalence of ABI in the PICU, the rate exhibited a fluctuating pattern from 2014 to 2022. From 2014 to 2016, the rate remained stable at 3.00, followed by a noticeable increase in 2017 to 4.14. The prevalence peaked in 2018 at 5.94, before slightly declining to 5.22 in 2019. A more significant decrease was observed in 2020 and 2021, with rates dropping to 3.97 and 3.78, respectively. However, in 2022, the prevalence rebounded to 5.4, approaching the 2018 peak. This overall trend suggests periodic fluctuations in ABI, with notable peaks occurring in 2018 and 2022.”

       This data is interesting and valuable for integration and analysis in this study. To further enhance the manuscript, consider incorporating graphs or illustrations to better visualize the findings, along with a more in-depth discussion to provide additional context and insights.  

 5. Please include additional information in the background section, focusing on key risk factors and reviewing relevant statistics. This will help readers better understand the context and significance of the findings.

 6. A discussion on drug resistance should also be included as part of the result and analysis. Lastly, ensure that a section on the limitations of the study is added to provide a more comprehensive view of the research.

Is the work clearly and accurately presented and does it cite the current literature?

Partly

If applicable, is the statistical analysis and its interpretation appropriate?

Partly

Are all the source data underlying the results available to ensure full reproducibility?

Yes

Is the study design appropriate and is the work technically sound?

Partly

Are the conclusions drawn adequately supported by the results?

Partly

Are sufficient details of methods and analysis provided to allow replication by others?

Partly

Reviewer Expertise:

infectious, HIV EID IPC and children

I confirm that I have read this submission and believe that I have an appropriate level of expertise to confirm that it is of an acceptable scientific standard, however I have significant reservations, as outlined above.

# Response

**Published:** 2025-04-24

**Authors:** Pornumpa Bunjoungmanee

**Affiliations:** pediatric, Thammasat University, Pathum Thani, Thailand

Thank you very much for your thoughtful review and valuable suggestions, which have significantly contributed to improving the quality of our manuscript. For clarity, we have included your original comments below, followed by our point-by-point responses in bold.

 1. This case-control study compares the risk factors for *Acinetobacter baumannii* infections with those for non- *Acinetobacter baumannii* infections (control group). While the information provided is valuable, further consideration of several issues is necessary, such as the selection criteria and characteristics of the control group, potential confounding factors, and the robustness of the data analysis.

         -A study by Uwingabiye J et al reported that *Acinetobacter baumannii* accounted for 22.6% of infections in the ICU. In several study, the mortality rate associated with *A. baumannii* infections ranges from 26.5% to 91%, reaching as high as 70% for infections caused by resistant strains. These findings highlight the severity of cases involving *A. baumannii*.

       - The selection of a control group is a critical aspect of case-control study design. In this study, the control group was defined as patients with non- *Acinetobacter baumannii* infections, representing less severe cases with fewer underlying conditions. To address this issue, the authors might consider selecting a more appropriate control group for comparison. For example, other studies have compared children with carbapenem-resistant *A. baumannii* (CRAB) infections to those with carbapenem-susceptible *A. baumannii* (CSAB) infections, which could provide a more balanced and clinically relevant comparison.

        The authors may consider matching the control group by adding specific conditions to ensure appropriate integration and analysis. This approach could help create a more balanced comparison and improve the validity of the findings.

**Ans:**

**In the initial version of the article, cases and controls were selected from patients admitted to the pediatric intensive care unit (PICU) during the same period, with a minimum stay of 48 hours. Random matching was conducted in a 1:2 ratio. The exclusion criteria included pediatric patients whose culture reported grew *Acinetobacter baumannii* within 48 hours of PICU admission or those with incomplete medical records. However, this approach posed a risk of potential confounding.**

**To address this issue, additional exclusion criteria were included: a minimum Pediatric Logistic Organ Dysfunction-2 (PELOD-2) score of 10 and an extended minimum PICU stay of 120 hours. These modifications were implemented to reduce confounding in the selection of case and control groups. A PELOD-2 score of ≥10 is considered a medium score, and is associated with an estimated mortality risk of approximately 10%. ^1-2 ^The researchers consider this threshold appropriate for identifying patients eligible to participate in the study.**

**Reference **

1. **Leteurtre S, Duhamel A, Salleron J, et al. PELOD-2: An Update of the PEdiatric Logistic Organ Dysfunction Score. Crit Care Med. 2013;41(7):1761-73. **
2. **López MPA, Prata-Barbosa A, Lima-Setta F. Severity of illness scores in the pediatric intensive care unit: a practical guide. Crit Care Sci. 2024 Oct 11;36:e20240205en.**

2. Case group: The Acinetobacter baumannii group is divided into two subgroups: resistant and non-resistant strains. Please consider this distinction, as several factors may differ between these subgroups and could influence the analysis and outcomes.

**Ans**

**In this study, *Acinetobacter baumannii* exhibited a carbapenem resistance rate of 94.26%, whereas the non-resistant group accounted for only 6.78%. As a result, the sample sizes for comparison were insufficient, limiting the ability to perform a valid comparative analysis.**

 3. If the authors intend to compare two groups for factor analysis, they should consider the sample size in each group to ensure sufficient statistical power and reliable results.

**Ans**

**Previous studies investigating *Acinetobacter baumannii* infections in the pediatric intensive care unit (PICU) have reported prevalence rates ranging from 10% to 20%. Notable studies include:**

**Katragkou A. et al., who conducted a study in the pediatric ICU in Greece from July 2001 to December 2003, reporting a prevalence of A. baumannii infection at 10.20%.**

**Reddy D. et al., who studied the pediatric ICU in South Africa in 2010 and found a prevalence of A. baumannii infection at 15.30%.**

**In this study, the estimated prevalence of** *A. baumannii* **infection in the pediatric ICU is consistent with prior reports, ranging from approximately 10% to 20%.**

**The sample size calculation was performed using the one-sample comparison of proportions formula in STATA version 14. The study was designed with a power of 90%, an alpha value of 0.05, and a two-sided test. A p-value of less than 0.05 was considered statistically significant. Based on the calculation in STATA, a minimum of 169 patients were required for inclusion. Given that a potential data collection deviation of approximately 20% might occur, the total number of participants included in the study was 210 individuals. The sample was selected using concurrent control sampling at a case-to-control ratio of 1:2, with randomization conducted via computer. Accordingly, the study included 70 cases and 140 controls.**

 4. This information demonstrated” Upon analyzing the annual prevalence of ABI in the PICU, the rate exhibited a fluctuating pattern from 2014 to 2022. From 2014 to 2016, the rate remained stable at 3.00, followed by a noticeable increase in 2017 to 4.14. The prevalence peaked in 2018 at 5.94, before slightly declining to 5.22 in 2019. A more significant decrease was observed in 2020 and 2021, with rates dropping to 3.97 and 3.78, respectively. However, in 2022, the prevalence rebounded to 5.4, approaching the 2018 peak. This overall trend suggests periodic fluctuations in ABI, with notable peaks occurring in 2018 and 2022.”

       This data is interesting and valuable for integration and analysis in this study. To further enhance the manuscript, consider incorporating graphs or illustrations to better visualize the findings, along with a more in-depth discussion to provide additional context and insights. 

**Ans**

**A graph** **has been incorporated into the study as per the recommendations. A detailed discussion of the changes in prevalence over the years has been included in the Discussion section.**

 5. Please include additional information in the background section, focusing on key risk factors and reviewing relevant statistics. This will help readers better understand the context and significance of the findings.

**Ans**

**A comprehensive overview of the risk factors for *Acinetobacter baumannii* infection in both adult and pediatric populations has been provided in the background section of the manuscript.**

 6. A discussion on drug resistance should also be included as part of the result and analysis. Lastly, ensure that a section on the limitations of the study is added to provide a more comprehensive view of the research.

**Ans**

**The antimicrobial resistance profile of *Acinetobacter baumannii* was presented in the results section, and the reasons for the resistance were discussed in the discussion section. Additionally, the limitations of the study were addressed in the strengths and limitations section.**
